# In Vitro Screening of Antibacterial Efficacy of *Moringa oleifera* and *Thymus vulgaris* Methanolic Extracts Against Different *Escherichia coli* Strains and Their In Vivo Effects Against *E. coli*-Induced Infection in Broiler Chickens

**DOI:** 10.3390/vetsci12100957

**Published:** 2025-10-06

**Authors:** Majid Ali, Naila Chand, Sarzamin Khan, Rifat Ullah Khan, Babar Maqbool, Shabana Naz, Ala Abudabos, Abdul Hafeez, Ibrahim A. Alhidary

**Affiliations:** 1 Department of Poultry Science, Faculty of Animal Husbandry and Veterinary Sciences, The University of Agriculture, Peshawar 25130, Pakistan; majidaup1@gmail.com (M.A.);; 2 Physiology Lab, College of Veterinary Sciences, Faculty of Animal Husbandry and Veterinary Sciences, The University of Agriculture, Peshawar 25130, Pakistan; 3 Department of Veterinary Medicine, Faculty of Veterinary & Animal Sciences, The University of Agriculture, Dera Ismail Khan 29163, Pakistan; babar.maqbool@uad.edu.pk; 4 Department of Zoology, Government College University, Faisalabad 38000, Pakistan; 5 Department of Food and Animal Sciences, College of Agriculture, Tennessee State University, Nashville, TN 37209, USA; 6 Department of Animal Production, College of Food and Agriculture Science, King Saud University, Riyadh 11451, Saudi Arabia

**Keywords:** *Moringa oleifera*, *T. vulgaris*, broiler chickens, *E. coli O78*, growth performance, gut health

## Abstract

**Simple Summary:**

This study investigated the antibacterial and growth-promoting effects of *Moringa oleifera* and *Thymus vulgaris* methanolic extracts in broiler chickens challenged with *Escherichia coli* O78. In vitro screening showed that ciprofloxacin was the most effective antibiotic, while *T. vulgaris* extract outperformed *M. oleifera*, with the best inhibition observed from their combination (M100T150). In vivo, addition of M100T150 improved weight gain, feed efficiency, and economic indices, while reducing gut pH and *E. coli* counts and increasing *Lactobacillus* populations. Histological examination confirmed improved villus structure and gut integrity in supplemented birds. These results highlight the potential of *M. oleifera* and *T. vulgaris* extracts as natural alternatives to antibiotics in broiler production.

**Abstract:**

This study evaluated the antibacterial efficacy and growth-promoting potential of *Moringa oleifera* and *Thymus vulgaris* methanolic extracts in broiler chickens challenged with *Escherichia coli* O78. In vitro antibacterial screening using agar well diffusion and disc diffusion assays revealed that ciprofloxacin exhibited the strongest inhibitory effect, followed by tetracycline and enrofloxacin, whereas among plant extracts, *T. vulgaris* was more effective than *M. oleifera*. The optimal combination (M100T150; 100 mg *M. oleifera* + 150 mg *T. vulgaris*) produced the largest inhibition zones against *E. coli* strains. For the in vivo trial, 540 Ross-308 broiler chicks were distributed into six treatment groups in a completely randomized design and reared for 42 days. Parameters assessed included growth performance, carcass traits, gut pH, ileal microbial counts, and intestinal histomorphology. Results showed that *E. coli* challenge significantly reduced feed intake, weight gain, carcass yield, and villus integrity while increasing FCR and *E. coli* counts (*p* < 0.05). Addition of plant extracts, particularly M100T150, significantly improved weight gain, FCR, Broiler Performance Efficiency Factor (BPEF), and Broiler Farm Economy Index (BFEI) compared to the positive control (*p* < 0.05). Extracts reduced duodenal and jejunal pH (*p* < 0.001), suppressed *E. coli* counts (*p* = 0.003), and enhanced *Lactobacillus* populations (*p* = 0.0004). Histological analysis revealed that extract-supplemented groups had greater villus height and surface area with shallower crypts than the positive control, indicating restoration of gut integrity. These findings suggest that methanolic extracts of *M. oleifera* and *T. vulgaris*, particularly in combination, can serve as natural alternatives to antibiotics in broiler production under pathogenic challenge.

## 1. Introduction

Avian colibacillosis, caused by avian pathogenic *Escherichia coli* (APEC), remains one of the most economically important bacterial diseases in the poultry industry worldwide [1]. The infection typically begins as septicemia and may progress to sudden death or localized inflammation in vital organs depending on the virulence of the strain. Host susceptibility and environmental conditions also influence disease outcome [2]. The widespread use of antibiotics in poultry—both for therapeutic and non-therapeutic purposes—has contributed to the rapid emergence of multidrug-resistant *E. coli* strains [3,4,5]. Although antibiotics remain a cornerstone in controlling bacterial infections, their overuse increases cost, results in residual toxicity in animal products, and accelerates antimicrobial resistance, thereby limiting treatment efficacy [6,7,8]. With resistance becoming a global public health concern, there is increasing interest in safe and sustainable alternatives to antibiotics, particularly plant-based compounds [9,10,11,12].

In this context, fluoroquinolones (ciprofloxacin, enrofloxacin) and tetracyclines were chosen in the present study as reference antibiotics because they are among the most widely used classes in poultry production and are critically important in managing colibacillosis. Their frequent use has been directly linked to the emergence of multidrug-resistant *E. coli* in poultry farms, making them highly relevant for evaluating the comparative efficacy of plant-derived antimicrobials. β-lactams were not included because they are not commonly used in poultry in the study region, allowing the design to focus on the most practical therapeutic challenges. By focusing on these antibiotic classes rather than others, such as β-lactams, the study better reflects the practical challenges faced in poultry health management.

Medicinal plants produce a wide range of secondary metabolites such as flavonoids, terpenoids, tannins, alkaloids, and essential oils, many of which exhibit potent antimicrobial, antioxidant, and immunomodulatory properties [13,14,15,16]. These phytochemicals disrupt microbial cell walls, alter membrane permeability, inhibit enzyme activity, and interfere with nucleic acid synthesis, thereby reducing bacterial growth and pathogenicity [17,18,19]. Moreover, the use of plant-based antimicrobials is considered safer, more eco-friendly, and less likely to contribute to resistance compared with conventional antibiotics [20,21].

Among the plants of current interest, *M. oleifera* has gained significant attention due to its broad pharmacological and nutritional benefits [17,22,23]. Extracts from its leaves, seeds, and flowers have demonstrated strong antibacterial activity, particularly against *E. coli* [24]. Beyond antimicrobial effects, *M. oleifera* leaves are rich in vitamins, minerals, and antioxidants, making them a dual-purpose feed additive that can promote growth, enhance immunity, and mitigate oxidative stress in poultry [22]. For example, dried leaves contain approximately 2.0–2.5 g calcium, 20–25 mg iron, and 150–200 mg vitamin C per 100 g of dry matter [25], highlighting their potential as a nutritionally functional feed component. Its higher calcium content than milk, higher iron than spinach, and higher vitamin C than oranges further highlight its value as a nutritionally functional feed component [25].

Similarly, *Thymus vulgaris* (thyme), a member of the Lamiaceae family, has been widely recognized for its medicinal and growth-promoting properties in animal production [26]. Its bioactivity is attributed to strong antibacterial, antifungal, anticoccidial, and antioxidant properties [27,28]. These compounds destabilize bacterial membranes, inhibit toxin production, and reduce intestinal pathogen load, thereby improving gut health and nutrient utilization. In poultry, thyme addition has been reported to enhance feed efficiency, reduce pathogenic *E. coli* counts, and improve overall performance [29].

Given the economic significance of colibacillosis and the limitations of antibiotic-based control strategies, there is a pressing need to explore natural plant-derived alternatives with proven antimicrobial efficacy. Both *M. oleifera* and *T. vulgaris* represent promising candidates, offering a synergistic combination of antimicrobial, antioxidant, and growth-promoting effects. This study aimed to evaluate the antibacterial efficacy of methanolic extracts of *Moringa oleifera* and *Thymus vulgaris* against pathogenic *E. coli* strains in vitro, and to determine their in vivo effects on performance, carcass quality, gut pH, microbial populations, and intestinal morphology under *E. coli* challenge.

## 2. Materials and Methods

### 2.1. Preparation of M. oleifera and T. vulgaris Methanolic Extracts

Fresh leaves of *Moringa oleifera* and *Thymus vulgaris* were collected from local areas of Khyber Pakhtunkhwa, Pakistan, and taxonomically authenticated by the Department of Horticulture, The University of Agriculture, Peshawar (voucher specimen Nos. MO-KP-2022-01 and TV-KP-2022-02, deposited in the departmental herbarium). Young green leaves of both plants were air-dried under shade to avoid direct exposure to sunlight, with regular turning to prevent fungal growth. After five days of drying, the leaves were ground using an electric grinder (Model BB90E) and sieved through a 0.15 mm mesh (Endecotts Ltd., London, UK) to obtain fine powders. For each plant, 400 g of powdered leaves were extracted with 2000 mL of absolute methanol (99.8%) in a Soxhlet apparatus. The mixtures were allowed to stand for 72 h, filtered initially through double-layered muslin cloth and subsequently through Whatman No. 1 filter paper following the method of Olayaki et al. [30]. The filtrates were concentrated using a rotary evaporator operated at 150 rpm and 35 °C to obtain the final methanolic extracts of *M. oleifera* and *T. vulgaris* [31]. Before use, the extracts were subjected to a preliminary phytochemical screening to confirm the presence of major bioactive constituents (flavonoids, phenolics, tannins, alkaloids, and saponins) using standard qualitative assays [1]. Quantitative determination of total phenolics and flavonoids was also performed (Folin–Ciocalteu and aluminum chloride methods, respectively) to provide a basic chemical profile of the preparations [31]. The sterile extracts (0.22 µm syringe-filtered) were stored at 4 °C until use.

### 2.2. Antibacterial Screening

**Figure 1:**
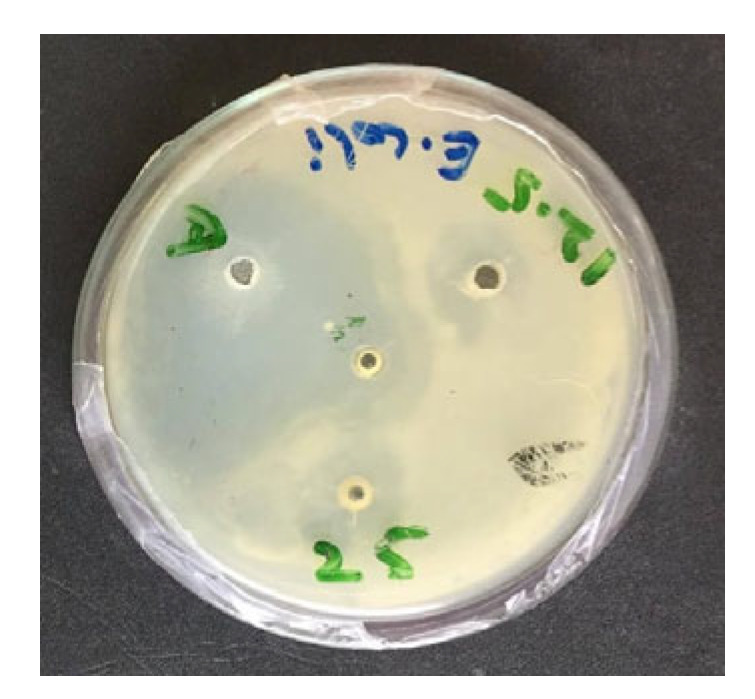
Antibacterial activity of methanolic extracts of *Moringa oleifera* and *Thymus vulgaris.*

The antibacterial efficacy of the methanolic extracts of *M. oleifera* and *T. vulgaris* against *Escherichia coli* (*E. coli* serotypes O78, O126, and O157) was evaluated using the agar well diffusion method under aerobic conditions. All strains were obtained as reference cultures from the National Veterinary Laboratory (NVL), Islamabad, Pakistan. Each strain was inoculated into 10 mL sterile nutrient broth and incubated at 37 °C for 8 h under aerobic conditions. Cultures were swabbed onto nutrient agar plates, and 6 mm wells were prepared with a sterile cork borer. Wells were filled with 100 µL of plant extract at concentrations of 25, 50, and 100 mg/mL, and plates were incubated overnight at 37 °C under aerobic conditions. Zones of inhibition (ZI) were measured after 24 h with a vernier caliper as described by Mona et al. [32]. In parallel, antibiotic sensitivity of the three *E. coli* strains was tested using the disc diffusion method on Mueller–Hinton agar with commercial discs of enrofloxacin, ciprofloxacin, and tetracycline (Oxoid, Basingstoke, UK) [33,34] as show in Figure 1.

### 2.3. Preparation of E. coli Inoculum and Challenge to Broiler Chickens

A freshly cultured *E. coli* O78 reference strain (NVL, Islamabad, Pakistan) was used for in vivo challenge. A standardized suspension of 1 × 10^9^, CFU/mL was prepared in sterile phosphate-buffered saline, and each bird received an oral gavage of 1 mL of this suspension (equivalent to 1 × 10^9^, CFU per bird) on a single occasion on day 15 of age. Birds in the negative control group received an equal volume of sterile PBS.

### 2.4. Experimental Design and Treatments Protocol

A total of 540 day-old Ross-308 broiler chicks were randomly allocated in a completely randomized design (CRD) to six treatment groups: negative control, positive control, standard, M100T150, M50T75, and M150T225. Each group was further divided into six replicates with 15 chicks per replicate. All birds were fed a basal diet formulated to meet nutrient specifications (Table 1). Birds were reared for 42 days and fed a basal corn–soybean diet formulated to meet or exceed nutrient requirements for starter (1–21 days), and finisher (22–42 days) phases. On day 15, all groups except the negative control were orally challenged with *E. coli* O78 at 1 × 10^9^ CFU. The negative control group remained unchallenged and untreated, while the positive control group was challenged but left untreated. The standard group was treated with ciprofloxacin (the most effective antibiotic identified in the sensitivity test) at 20 mg/L in drinking water. Group M100T150 received the optimal combination level of *M. oleifera* (100 mg) and *T. vulgaris* (150 mg) extracts identified in Experiment I, whereas groups M50T75 and M150T225 were treated with 50 and 75 mg and 150 and 225 mg of this combination level, respectively, in drinking water. Chicks in all groups were reared under optimal conditions, including proper temperature, feeders, drinkers, and management practices, to ensure full genetic potential expression. The trial lasted for 42 days, including a 7-day adaptation period. All management practices (temperature, feeders, and drinkers) followed commercial standards to maximize growth performance.

### 2.5. Growth Performance and Carcass Characteristics

Growth performance of broilers was evaluated through feed intake, body weight gain, feed conversion ratio (FCR), livability, broiler performance efficiency factor (BPEF), broiler farm economy index (BFEI), and dressing percentage. Feed intake was recorded daily by subtracting the refused feed from the total feed offered, while weekly body weight gain was determined as the difference between the initial and final body weights of birds for each week. The FCR was calculated weekly as the ratio of feed intake to body weight gain. Livability was expressed as the percentage of surviving birds at the end of the experiment relative to the initial number of birds at the start. The BPEF, an economic indicator of flock performance, was computed by dividing live body weight (g) by FCR and multiplying by 100. Similarly, the BFEI was determined using the formula: (average live weight × percentage livability)/(FCR × growing period in days). Dressing percentage was assessed by randomly selecting five birds per replicate, recording their live weights, and calculating carcass yield after removal of skin and inedible parts. Edible cuts such as thigh, leg, breast, and wings were weighed, and dressing percentage was expressed as the ratio of carcass weight to live weight multiplied by 100.

### 2.6. Gut pH

The pH of different segments of the gastrointestinal tract was measured using an electronic pen-type pH meter. At the time of slaughtering, the electrode was carefully inserted into organs containing chyme and digesta, and pH values were recorded for the crop, gizzard, and small intestine [35].

### 2.7. Ileal Microbial Count

On day 42 of the trial, two birds per replicate were slaughtered, and digesta samples were collected from the ileal region. For microbial enumeration, three samples from each replicate of each group were pooled and analyzed. One gram of digesta was diluted in 9 mL of 1% peptone solution and gently homogenized, after which serial 10-fold dilutions were prepared. The diluted samples were plated onto de Man, Rogosa, and Sharpe (MRS) agar for *Lactobacillus* and MacConkey agar for *E. coli* isolation. Plates were incubated at 37 °C for 24 h and bacterial colonies were counted immediately as described by Deris et al. [36].

### 2.8. Intestinal Histomorphology

Histological examination of the ileum was carried out on day 42 of the experimental trial. Two birds per replicate were slaughtered, and ileal segments (approximately 2 cm in length) were excised from the midpoint between Meckel’s diverticulum and the ileocecal junction. Tissues were gently flushed with physiological saline solution to remove digesta, and samples were immediately fixed in 10% buffered formalin for 48 h. After fixation, tissues were dehydrated through graded ethanol series, cleared in xylene, and embedded in paraffin wax. Sections of 5 μm thickness were cut using a microtome and mounted on glass slides. The slides were stained with hematoxylin and eosin (H&E) and examined under a light microscope fitted with a calibrated eyepiece micrometer.

Morphometric parameters, including villus height (measured from the tip of the villus to the villus–crypt junction), villus width (measured at the midpoint of the villus), crypt depth (measured from the base of the villus to the submucosa), villus height-to-crypt depth ratio, and villus surface area (calculated as 2π × (villus width/2) × villus height), were determined. For accuracy, ten well-oriented villi and crypts were measured per slide, and mean values were used for statistical analysis.

### 2.9. Statistical Analysis

Data were analyzed using one-way analysis of variance (ANOVA) to evaluate the effect of different combinations of *M. oleifera* and *T. vulgaris* methanolic extracts on carcass traits, microbial counts, and intestinal histomorphology of broiler chickens challenged with *E. coli* O78. Treatment means were compared using Tukey’s post hoc test, and results were expressed as mean ± standard error of the mean (SEM). Differences among treatment groups were considered statistically significant at a probability level of *p* < 0.05.

## 3. Results

### 3.1. In Vitro Antibacterial Activity

The in vitro antibacterial activities of the methanolic extracts are summarized in Table 2. Ciprofloxacin produced the largest inhibition zones against all three *Escherichia coli* strains, followed by tetracycline and enrofloxacin. Among plant extracts, Thymus vulgaris produced larger inhibition zones than Moringa oleifera. The combination of *M. oleifera* (100 mg mL^−1^) with *T. vulgaris* (150 mg mL^−1^) produced larger zones than any other plant-extract treatment (*p* < 0.05 vs. all other combinations). Inhibition differed among *E. coli* serotypes: O78 showed larger zones than O126 (*p* = 0.003) and O157 (*p* = 0.015).

### 3.2. Growth Performance (0–42 Days)

Cumulative feed intake was higher in the negative control than in the positive control (*p* < 0.001) and did not differ from the standard-antibiotic or M100T150 groups (Table 3). Body-weight gain was higher in the negative control compared with the positive control (*p* < 0.001); M100T150 and the standard group achieved gains comparable to the negative control and greater than M50T75 or M150T225 (*p* < 0.05). Feed-conversion ratio (FCR) was lower (better) in the negative control and M100T150 groups compared with the positive control (*p* < 0.001). Livability was similar among all treatments (*p* = 0.074). Broiler Performance Efficiency Factor and Broiler Farm Economy Index were higher in the negative control and M100T150 groups than in the positive control (*p* < 0.001 and *p* = 0.0025, respectively).

### 3.3. Carcass Characteristics

Dressing percentage was higher in the negative control than in the positive control (*p* = 0.0003), with intermediate values in extract-supplemented and standard groups (Table 4). Thigh yield was higher in the negative control than in the positive control (*p* = 0.0015). Breast yield was higher in the negative control than in the positive control (*p* = 0.0045); extract treatments produced intermediate values. Wing yield did not differ among groups (*p* = 0.72).

### 3.4. Gastrointestinal pH

Crop and gizzard pH did not differ among treatments (*p* = 0.94 and *p* = 0.67; Table 5). Duodenal, jejunal, and ileal pH were lower in the M100T150 group than in the positive control (all *p* < 0.001) and also lower than in the negative control for the duodenum and jejunum (*p* < 0.05).

### 3.5. Ileal Microbial Counts

Ileal *E. coli* counts were lower in all extract-supplemented groups and the standard group compared with the positive control (*p* = 0.003), with the lowest counts in the negative control and M100T150 (Table 6). Lactobacillus counts were higher in the M100T150 and M50T75 groups than in the positive control (*p* = 0.0004).

### 3.6. Intestinal Histomorphology

**Figure 2:**
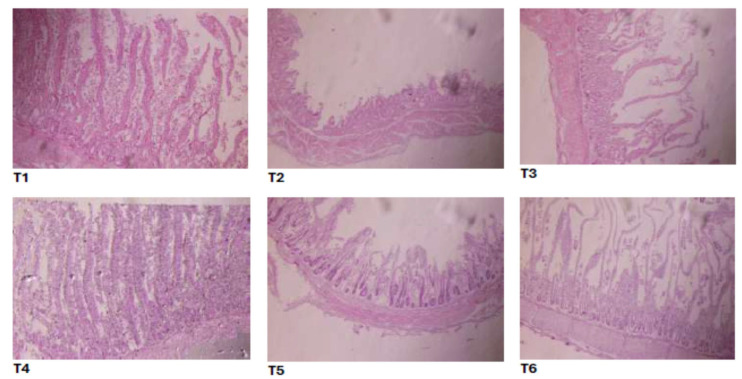
Effect of different combinations of *M. oleifera* and *T. vulgaris* methanolic extracts on intestine histopathology of broiler challenged with *E. coli O78.* **T1—Negative control** (unchallenged, no extract), **T2—Positive control** (*E. coli*–challenged, no extract), **T3—Standard antibiotic control**, **T4—M50T75** (*M. oleifera* + *T. vulgaris* at 50 and 75 mg/L), **T5—M100T150** (*M. oleifera* + *T. vulgaris* at 100 and 150 mg/L), and **T6—M150T225** (*M. oleifera* + *T. vulgaris* at 150 and 225 mg/L).

Villus height, villus width, crypt depth, and villus surface area differed among treatments (*p* = 0.0040, 0.0455, 0.0035, and 0.0225, respectively; Table 7 and Figure 2). The positive control showed the lowest villus height and surface area and the deepest crypts. The negative control, standard group, and M100T150 group had greater villus height and surface area and shallower crypts than the positive control (*p* < 0.05). Villus-height-to-crypt-depth ratio did not differ among treatments (*p* = 0.318).

## 4. Discussion

The present study evaluated the effects of *M. oleifera* and *T. vulgaris* methanolic extracts, all provided as different combination levels rather than individual single-plant treatments, on growth performance, carcass traits, gut health, and intestinal histomorphology of broilers challenged with *E. coli* O78. The findings demonstrated that the addition of the M100T150 combination improved growth and gut health compared with the positive control (*p* < 0.05), with the combined dose of 100 mg/L *M. oleifera* and 150 mg/L *T. vulgaris* consistently producing the best outcomes.

Growth performance data indicated a clear dose-dependent improvement, where the M100T150 group showed higher body-weight gain and better feed-conversion ratio than other extract combinations, suggesting a potential synergistic effect between the two extracts. Moringa contains flavonoids, phenolic acids, and glucosinolates with antioxidant and antimicrobial properties [37], whereas thyme provides thymol and carvacrol, potent phenolics with strong antibacterial activity [38]. When combined, these phytochemicals may act additively or synergistically to reduce oxidative stress, suppress enteric pathogens, and improve nutrient utilization.

Microbial analysis showed that *E. coli* counts were lower in M100T150 compared with the positive control (*p* = 0.003), declining from 8.67 to 5.57 log CFU/g. Because no direct time–kill or bactericidal assays were performed, this finding is described as antimicrobial activity rather than definitive bactericidal action. Future work should include standardized bactericidal testing to confirm this mechanism. Better growth in the extract-added groups compared with the positive control can be explained by several mechanisms. Infection with *E. coli* damages intestinal mucosa, reducing feed intake and nutrient absorption [39,40]. The extracts may counteract these effects by lowering pathogen load and permitting improved nutrient absorption. Phenolic compounds from thyme stimulate pancreatic and intestinal enzyme secretion [41], and Moringa flavonoids can enhance digestive enzyme activity and protein metabolism [42].

Carcass characteristics reflected similar trends: birds receiving M100T150 showed higher dressing percentage and breast yield compared with the positive control (*p* < 0.01), while groups with higher individual extract levels produced intermediate values, indicating that excessively high doses did not further improve carcass traits. Gut environment results showed that jejunal and ileal pH were lower in M100T150 compared with the positive control (*p* < 0.001), consistent with earlier reports that phytogenic compounds acidify the intestinal lumen [43,44,45]. A lower pH favors beneficial microbiota such as Lactobacillus spp. [46]. Indeed, *E. coli* counts were lower and Lactobacillus counts were higher in extract-added groups than in the positive control. These effects are plausibly linked to the ability of thymol and carvacrol to disrupt bacterial membranes [47] and isothiocyanates from *M. oleifera* that interfere with quorum sensing [37].

Histological observations supported these findings: villus height and villus surface area were higher and crypt depth was shallower in M100T150 compared with the positive control (*p* < 0.05), indicating enhanced absorptive capacity. Overall, the results agree with previous studies showing that phytogenic additives can improve growth, immunity, and gut health in broilers [48,49,50,51]. The present work highlights that moderate inclusion of a synergistic combination (M100T150) produced superior outcomes compared with higher inclusion levels, consistent with a biphasic dose–response where excessive concentrations may reduce benefits [51,52].

## 5. Conclusions

The methanolic extracts of *Moringa oleifera* and *Thymus vulgaris* demonstrated dose-dependent antibacterial, growth-promoting, and gut-protective effects in broilers challenged with *E. coli*, with the M100T150 combination consistently improving growth performance, carcass traits, gut pH, microbial balance, and intestinal histomorphology comparable to the standard antibiotic group. However, instead of repeating numerical outcomes, the key practical implication is that moderate inclusion of these plant extracts could serve as a potential alternative to in-feed antibiotics—provided that future work confirms their chemical composition, establishes reproducible bactericidal activity through standardized assays, and evaluates the cost–benefit of commercial use. Clear economic feasibility, long-term safety, and resistance risk assessments remain essential next steps before field application.

## Figures and Tables

**Table 1:** Composition of basal feed of experimental birds.

Ingredients (%)	Starter Phase (1–21 Day)	Finisher Phase (22–42) Day
Corn gluten meal	1.98	7.14
Soybean meal	37.6	24.0
Corn	54.1	60.2
Corn oil	2.18	2.70
Dicalcium phosphate	2.35	2.10
Limestone	0.81	0.67
Dicalcium phosphate	2.35	2.10
VM mix ^a^	0.50	0.50
Salt	0.50	0.55
Lysine HCL	0.21	0.40
Threonine	0.12	0.10
DL-Methionine	0.22	0.12
Choline chloride	0.06	0.06
Chemical composition		
Crude protein (%)	23.0	21.0
ME (Kcal/kg)	3000	3150
Methionine (%)	0.54	0.44
Sulphur amino acid (%)	0.9	0.78
Lysine (%)	1.40	1.22
Calcium (%)	1.06	0.90
Phosphorus (%)	0.50	0.46
Threonine (%)	0.95	0.88

^a^ Vitamin-mineral premix contains in the following per kg: vitamin A, 2,400,000 IU; vitamin D, 1,000,000 IU; vitamin E, 16,000 IU; vitamin K, 800 mg; vitamin B1, 600 mg; vitamin B2, 1600 mg; vitamin B6, 1000 mg; vitamin B12, 6 mg; niacin, 8000 mg; folic acid, 400 mg; pantothenic acid, 3000 mg; biotin 40 mg; antioxidant, 3000 mg; cobalt, 80 mg; copper, 2000 mg; iodine, 400; iron, 1200 mg; manganese, 18,000 mg; selenium, 60 mg, and zinc, 14,000 mg.

**Table 2:** In vitro antibacterial activities of methanolic extracts of *M. oleifera* and *T. vulgaris*, alone and in different combinations, against selected pathogenic strains of *E. coli.*

Group	Combination/Concentration	*E. coli* O78 Mean Zone of Inhibition (mm) ± SEM	*E. coli* O126 Mean Zone of Inhibition (mm) ± SEM	*E. coli* O157 Mean Zone of Inhibition (mm) ± SEM	Means (Combination)
Standard antibiotics	Enrofloxacin (20 µg/disc)	17.47 ± 0.12 ^b^	14.39 ± 0.34 ^b^	16.65 ± 0.11 ^b^	16.17
	Ciprofloxacin (20 µg/disc)	22.51 ± 0.34 ^a^	20.36 ± 0.88 ^a^	22.05 ± 0.35 ^a^	21.64
	Tetracycline (20 µg/disc)	18.45 ± 0.65 ^b^	17.93 ± 0.91 ^b^	19.79 ± 0.66 ^b^	18.72
	*p*-values	0.98	0.11	0.43	0.44
M100	*M. oleifera* (200 mg/mL)	9.40 ± 0.41 ^c^	1.44 ± 0.14 ^e^	4.15 ± 0.05 ^b^	5.00 ± 0.35 ^c^
T100	*T. vulgaris* (300 mg/mL)	10.15 ± 0.18 ^b^	6.70 ± 0.04 ^b^	3.50 ± 0.11 ^c^	6.78 ± 0.29 ^b^
M50T50	*M. oleifera* (100 mg/mL) + *T. vulgaris* (150 mg/mL)	13.70 ± 0.10 ^a^	8.50 ± 0.16 ^a^	5.15 ± 0.03 ^a^	9.12 ± 0.37 ^a^
M75T25	*M. oleifera* (150 mg/mL) + *T. vulgaris* (75 mg/mL)	9.55 ± 0.07 ^c^	2.30 ± 0.04 ^d^	2.45 ± 0.05 ^d^	4.77 ± 0.35 ^d^
M25T75	*M. oleifera* (50 mg/mL) + *T. vulgaris* (225 mg/mL)	7.50 ± 0.04 ^d^	2.75 ± 0.09 ^c^	1.75 ± 0.05 ^e^	4.00 ± 0.27 ^e^
Mean (*E. coli*)	–	10.06 ± 0.21 ^a^	4.34 ± 0.29 ^b^	3.40 ± 0.12 ^c^	–
*p*-values	–	0.025	0.003	0.015	–

The treatments in the same row with different superscripts are significantly different at a significance level of α = 0.05. Where M = *Moringa oleifera*; T = *Moringa oleifera.*

**Table 3:** Effects of different combinations of methanolic extracts of *M. oleifera* and *T. vulgaris* on production traits in broiler birds challenged with *E. coli O78.*

Groups	Feed Intake (g)	Weight Gain (g)	Feed Conversion Ratio	Livability (%)	Broiler Performance Efficiency Factor	Broiler Farm Economy Index
−Ve cont. (uninfected, untreated negative control)	3613.37 ^a^ ± 1.7	2174.94 ^a^ ± 5.56	1.66 ± 0.02 ^c^	100 ± 0.00	131 ± 4.26 ^a^	3.11 ± 0.10 ^a^
+Ve cont. (infected, untreated positive control)	3270.96 ^d^ ± 4.2	1623.61 ^d^ ± 3.7	2.01 ± 0.04 ^a^	73.99 ± 10.18	80.77 ± 4.01 ^c^	1.23 ± 0.19 ^d^
Standard	3549.36 ^b^ ± 1.41	2025.01 ^b^ ± 3.6	1.75 ± 0.05 ^bc^	88.88 ± 2.22	115.71 ± 0.57 ^b^	2.44 ± 0.08 ^b^
M100T150	3515.37 ^b^ ± 2.38	2053.64 ^b^ ± 12.9	1.69 ± 0.02 ^c^	88.88 ± 5.87	121.51 ± 2.69 ^a^	2.57 ± 0.18 ^b^
M50T75	3450.36 ^c^ ± 2.5	1968.64 ^bc^ ± 7.2	1.74 ± 2.90 ^bc^	75.55 ± 11.75	113.14 ± 0.24 ^b^	2.03 ± 0.32 ^c^
M150T225	3420.99 ^c^ ± 1.9	1887.34 ^c^ ± 1.5	1.81 ± 0.01 ^b^	82.22 ± 11.75	104.27 ^b^ ± 2.02	2.04 ^c^ ± 0.49
*p* value	0.001	0.001	0.001	0.0740	0.001	0.0025

Means in the same column with various superscripts for each treatment are significantly different at α = 0.05. M100T150 = *M. oleifera* + *T. vulgaris* at the rate of 100 and 150 mg, respectively; M50T75 = *M. oleifera* + *T. vulgaris* at the rate of 50 and 75 mg, respectively; M150T1225 = *M. oleifera* + *T. vulgaris* at the rate of 150 and 225 mg, respectively.

**Table 4:** Effect of different combinations of *M. oleifera* and *T. vulgaris* methanolic extracts on dressing organ of broiler birds challenged with *E.coli 078.*

Groups	Dressing (%)	Thigh (%)	Breast (%)	Wing (%)
Negative control	68.94 ^a^ ± 0.14	19.17 ± 0.46 ^a^	32.38 ± 0.23 ^a^	10.28 ± 0.95
Positive control	61.39 ^c^ ± 0.26	14.58 ± 0.21 ^d^	27.17 ± 0.10 ^c^	7.76 ± 1.41
Standard	65.15 ^b^ ± 0.13	16.96 ± 0.01 ^bc^	30.11 ± 0.37 ^b^	8.37 ± 0.57
M100T150	65.52 ^b^ ± 0.72	17.51 ± 0.05 ^b^	30.50 ± 0.29 ^b^	8.95 ± 1.32
M50T75	65.88 ^b^ ± 0.32	17.48 ± 0.58 ^b^	30.34 ± 0.45 ^b^	8.30 ± 0.89
M150T225	63.81 ^bc^ ± 0.42	15.67 ± 0.18 ^c^	28.18 ± 0.18 ^bc^	8.43 ± 0.77
*p* value	0.003	0.0015	0.0045	0.72

The treatments in the same column with different superscripts are significantly different at a significance level of α = 0.05. M100T150 = *M. oleifera* + *T. vulgaris* at the rate of 100 and 150 mg, respectively; M50T75 = *M. oleifera* + *T. vulgaris* at the rate of 50 and 75 mg, respectively; M150T1225 = *M. oleifera* + *T. vulgaris* at the rate of 150 and 225 mg, respectively.

**Table 5:** Effect of different combinations of *M. oleifera* and *T. vulgaris* methanolic extracts on gut pH of broiler challenged with *E. coli O78*.

Groups	Duodenum	Jejunum	Ileum	Crop	Gizzard
Negative control	6.21 ^a^ ± 0.13	7.12 ± 0.22 ^a^	7.47 ± 0.04 ^a^	5.63 ± 0.20	3.65 ± 0.20
Positive control	6.17 ^a^ ± 0.08	7.01 ± 0.15 ^a^	7.43 ± 0.04 ^a^	5.49 ± 0.26	3.61 ± 0.23
Standard	5.96 ^b^ ± 0.08	6.42 ± 0.29 ^b^	7.17 ± 0.35 ^bc^	5.69 ± 0.41	3.90 ± 0.14
M100T150	5.01 ^c^ ± 0.19	5.09 ± 0.08 ^c^	7.05 ± 0.08 ^c^	5.34 ± 0.37	3.95 ± 0.10
M50T75	5.79 ^b^ ± 0.24	6.41 ± 0.21 ^b^	7.31 ± 0.06 ^b^	5.79 ± 0.43	4.06 ± 0.40
M150T225	5.86 ^b^ ± 0.14	6.08 ^b^ ± 0.31	7.27 ^b^ ± 0.04	5.67 ± 0.17	3.91 ± 0.04
*p* value	0.001	0.001	0.001	0.93	0.66

The treatments in the same column with different superscripts are significantly different at a significance level of α = 0.05. M100T150 = *M. oleifera* + *T. vulgaris* at the rate of 100 and 150 mg, respectively; M50T75 = *M. oleifera* + *T. vulgaris* at the rate of 50 and 75 mg, respectively; M150T1225 = *M. oleifera* + *T. vulgaris* at the rate of 150 and 225 mg, respectively.

**Table 6:** Effect of different combinations of *M. oleifera* and *T. vulgaris* methanolic extracts on microbial count of broiler challenged with *E.coli 078*.

Groups	*E. coli* (log cfu/g)	*Lactobacillus* (log cfu/g)
Negative control	3.20 ± 0.02 ^d^	4.84 ± 0.06 ^b^
Positive control	8.67 ± 0.24 ^a^	4.28 ± 0.14 ^c^
Standard	5.17 ± 0.11 ^c^	4.74 ± 0.05 ^b^
M100T150	5.57 ± 0.23 ^c^	5.72 ± 0.14 ^a^
M50T75	6.21 ± 0.30 ^b^	5.48 ± 0.11 ^a^
M150T225	6.65 ± 0.17 ^b^	4.88 ± 0.06 ^b^
*p* value	0.003	0.0004

The treatments in the same column with different superscripts are significantly different at a significance level of α = 0.05. M100T150 = *M. oleifera* + *T. vulgaris* at the rate of 100 and 150 mg, respectively; M50T75 = *M. oleifera* + *T. vulgaris* at the rate of 50 and 75 mg, respectively; M150T1225 = *M. oleifera* + *T. vulgaris* at the rate of 150 and 225 mg, respectively.

**Table 7:** Effect of different combinations of *M. oleifera* and *T. vulgaris* methanolic extracts on intestine histopathology of broiler challenged with *E. coli O78*.

Group	Villus Height (µm)	Villus Width (µm)	Crypt Depth (µm)	Villus Height to Crypt Depth Ratio	Villus Surface Area (µm^2^)
Negative control	830 ± 4.08 ^a^	81 ± 0.30 ^a^	71 ± 0.53 ^c^	11.69 ± 0.34	211 ± 0.2 ^a^
Positive control	182 ± 1.21 ^d^	31 ± 0.32 ^d^	176 ± 1.23 ^a^	1.03 ± 0.18	17.71 ± 0.34 ^d^
Standard	728 ± 2.66 ^b^	67 ± 0.17 ^b^	91 ± 2.22 ^bc^	8.00 ± 0.26	153.15 ± 2.32 ^b^
M100T150	713 ± 1.31 ^b^	61 ± 0.11 ^b^	103 ± 2.78 ^b^	6.92 ± 0.43	136.56 ± 1.40 ^b^
M50T75	607 ± 0.41 ^c^	43 ± 0.22 ^c^	151 ± 2.22 ^ab^	4.01 ± 0.08	85.39 ± 0.12 ^c^
M150T225	612 ± 1.50 ^c^	49 ± 0.33 ^c^	147 ± 2.78 ^ab^	4.16 ± 0.17	94.16 ± 0.56 ^c^
*p* value	0.0040	0.0455	0.0035	0.318	0.0225

The treatments in the same row with different superscripts are significantly different at a significance level of α = 0.05. M100T150 = *M. oleifera* + *T. vulgaris* at the rate of 100 and 150 mg, respectively; M50T75 = *M. oleifera* + *T. vulgaris* at the rate of 50 and 75 mg, respectively; M150T1225 = *M. oleifera* + *T. vulgaris* at the rate of 150 and 225 mg, respectively.

## Data Availability

The original contributions presented in this study are included in the article. Further inquiries can be directed to the corresponding authors.
